# Discriminant validity and test re-test reproducibility of a gait assessment in patients with vestibular dysfunction

**DOI:** 10.1186/s12901-015-0019-8

**Published:** 2015-10-22

**Authors:** Annatina Schmidheiny, Jaap Swanenburg, Dominik Straumann, Eling D. de Bruin, Ruud H. Knols

**Affiliations:** Directorate Research and Education Office, Physiotherapy Occupational Therapy Research, University Hospital Zurich, Zurich, Switzerland; Institute of Physiotherapy, Zurich University of Applied Sciences, Winterthur, Switzerland; Department of Physiotherapy, Balgrist University Hospital Zurich, Zurich, Switzerland; Department of Chiropractic Medicine, Faculty of Medicine, Balgrist University Hospital, Zurich, Switzerland; Department of Neurology, University Hospital, Zurich, Switzerland; Department of Health Sciences and Technology, Institute of Human Movement Sciences and Sport, ETH Zurich, Zurich, Switzerland; Centre for Evidence Based Physiotherapy, Maastricht University, PO Box 616, Maastricht, 6200 MD The Netherlands; Department of Epidemiology, CAPHRI School for Public Health and Primary Care, Maastricht University, PO Box 616, Maastricht, 6200 MD The Netherlands

**Keywords:** Vestibular diseases, Gait analysis, Validity, Reliability

## Abstract

**Background:**

Gait function may be impaired in patients with vestibular disorders, making gait assessment in the clinical setting relevant for this patient population. The purpose of this study was to evaluate the discriminant validity of a gait assessment protocol between patients with vestibular disorders and healthy participants. Furthermore, test re-test reproducibility and the measurement error of gait performance measures in patients with vestibular lesions was performed under different walking conditions.

**Methods:**

Gait parameters of thirty-five patients with vestibular disorders and twenty-seven healthy controls were assessed twice with the GAITRite® system. Discriminant validity, reproducibility (intra class correlation [ICC]) and the measurement error (standard error of measurement [SEM], smallest detectable change [SDC]) were determined for gait speed, cadence and step length. Bland-Altman plots were made to assess systematic bias between tests.

**Results:**

A significant effect of grouping on gait performance indicates discriminant validity of gait assessment. All tests revealed differences between patients and healthy controls (*p* < 0.01). The ICCs for test re-test reproducibility were excellent (0.70-0.96) and measurement error showed acceptable SDC values for gait parameters derived from three walking conditions (9-19 %). Bland-Altman plots indicated no systematic bias.

**Conclusions:**

Good validity and reproducibility of GAITRite® system measurements suggest that this system could facilitate the study of gait in patients with vestibular disorders in clinical settings. The SDC values for gait are generally small enough to detect changes after a rehabilitation program for patients with vestibular disorders.

## Background

Patients with vestibular disorders typically suffer from vertigo, vision disorders, body imbalance and limitation in mobility and the activities of daily living [1, 2]. Vertigo symptoms are usually triggered by activities that require head movements and transfers, or during walking [3, 4]. Furthermore, vestibular dysfunction is an important predictor for falls, especially in aging people [5]. The prevalence of dizziness and vertigo in Europe is 20—30 % in adults, of which approx. 7.8 % are defined as having a vestibular vertigo [6, 7].

The current management of vestibular disorders includes vestibular rehabilitation, pharmacological treatment, surgery, manual therapy and positioning manoeuvres for a specific diagnostic group of benign paroxysmal positional vertigo [8–10]. To date, there is moderate to strong evidence for vestibular rehabilitation to be effective in the management of unilateral peripheral vestibular dysfunction for improving balance and walking skills [8].

Specific walking parameters of patients with vestibular disorders need to be assessed for diagnosis and reassessment after therapy. For this reason, valid and reliable instruments monitoring patients' gait are required. Gait abnormalities may be assessed with the Functional Gait Assessment (FGA), a 10-item assessment based on the Dynamic Gait Index [11]. Although the FGA is a practical and functional assessment tool, it does not quantify temporal and spatial gait parameters beyond a sum score. Quantification of gait parameters while performing the FGA would, however, add more sophisticated information to a gait assessment.

Laboratory-based measurement instruments have been developed to enable improvement of walking analyses and to document diagnostic and therapeutic effectiveness. For instance, the GAITRite® walkway analysis system was developed to measure temporal and spatial gait parameters by using an approximate seven-metre walkway embedded with pressure sensors. This provides objective, precise and repeatable measurements in various clinical populations [12, 13]. The GAITRite® walkway analysis system was used in several studies and showed good validity and reliability for measuring temporal and spatial gait parameters in healthy adults [14]. Previous research tested the GAITRite® system in young and elderly (healthy) participants and patients with Parkinson's disease and stroke [13, 15, 16]. Schniepp, et al. [17] determined the variability of gait parameters using the GAITRite® system in patients with cerebellar ataxia, patients with vestibular disorders and healthy participants. Self-selected walking speed for healthy participants was 1.11 ± 0.19 m/s, for cerebral ataxia 1.0 ± 0.2 m/s and for patients with bilateral vestibular disorders 1.0 ± 0.2 m/s, indicating a difference of approximately 10 % between healthy participants and patients with vestibular disorders.

When a novel instrument is introduced for clinical use in a patient population it is important to evaluate the degree to which scores of different relevant groups deviate with a feasible measurement protocol [18]. Thus; publication of study results will establish the stability of an assessment. We hypothesised that gait assessed with the GAITRite® system would reveal differences for self-selected walking speed, cadence and step length between patients with vestibular disorders and healthy age-matched adults [13].

Furthermore, to be clinically meaningful, the measurement procedure also needs to be reliable in detecting differences in outcomes after a therapeutic intervention [19]. Reliability can be reported in terms of reproducibility [20, 21], which indicates the degree of association between two or more measures (e.g. Intraclass Correlation Coefficients [ICC]) [20], but does not provide clinical guidance for assessing true changes in individuals [22, 23].

Several studies evaluated the psychometric properties of the GAITRite® system and demonstrated good reproducibility. Hollman, et al. [24] reported excellent ICCs for velocity and cadence with ICC_ (2.1)_ values >0.84 in older people under single and dual-task walking conditions.

In stroke patients, test re-test reproducibility measures for the GAITRite® system were consistent with ICC _(2.1)_ values varying from 0.72 to 0.98 [16, 25].

Measurement error reflects the differences between two measures [26]. Examples of these measures are the standard error of measurement [SEM], calculated as the square root of error variance and the smallest detectable change [SDC] [27, 28]. To be clinically useful, measurement error needs to be considered in relation to meaningful change or clinically important differences [29].

In daily routine the assessment of gait patterns, with or without additional motor or cognitive tasks (dual tasking, such as counting backwards while walking or rotating the head, while walking), are common in clinical practice, e.g. diagnostic investigations in patients with vestibular disorders [30–32]. These clinical protocols do not, however, provide detailed information on changes in distinctive aspects of gait parameters that have potential clinical importance. Given the selective response of gait to pathology and evolution of disease, a more selective approach that would allow the observation of important changes in gait parameters is required [33]. Thus, when evaluating the validity and reliability of a gait assessment protocol, additional motor tasks should also be considered together with techniques that allow more detailed parameters of gait. Based on the FGA, a gait protocol was developed to evaluate 7 of 10 FGA tasks with the GAITRite® system.

In this study, the hypothesis was tested if the GAITRite® system could discriminate patients with vestibular disorders from healthy participants for the outcomes of self-selected walking speed, cadence and step length. Based on the study of Menz, et al. [13], a magnitude of 10 % or larger difference in outcomes was defined. Further, patients were evaluated twice to determine the test re-test reproducibility (ICC 2.1) and the measurement error (SEM, SDC) of walking behaviour as assessed with the GAITRite®. We conducted this study to (a) investigate the degree to which the scores of a gait analysis performed with the GAITRite® differ between patients with vestibular disorders and healthy participants, (b) identify the reproducibility of gait parameters measured with GAITRite® in patients with vestibular disorders walking under single and dual-task conditions, and (c) identify the measurement error (precision).

## Methods

### Design

A cross-sectional study design was chosen.

### Patients and participants

The study sample included patients with vestibular disorders and healthy control subjects. Patients with a diagnosis of vestibular dysfunction undergoing neuro-otological investigation at the University Hospital Zurich, Switzerland were recruited from the Departments of Neurology and Otorhinolaryngology at the Hospital. The vestibular testing battery of patients included three-dimensional video or search-coil head impulse testing along all 6 semicircular canals, caloric warm and cold water testing of both ears, subjective visual vertical, as well as ocular and cervical vestibular evoked myogenic potentials. For the purpose of this study, however, the definition of a vestibular deficit relied only on the functions of the horizontal semicircular canals, as assessed with horizontal head impulse testing to both sides (video or search coil system) [34–36] and caloric irrigation (video-oculography) [37]. Healthy subjects were recruited by personal invitation, e-mail and flyer from the staff of the University Hospital Zurich and from community dwellers in the greater area of Zurich. To be included in the study, participants were required to be aged over 18 years, subjects from the patient group needed to be diagnosed with a vestibular disorder. The following participants were excluded: after successful re-positioning manoeuvres compensating the vertigo symptoms, if they were not able to walk ten meters independently, had acute pain, uncontrolled cardiovascular disease, hip or knee endo-prosthesis, weakness due to neurological problems, or being known as or suspected of being non-compliant. All study participants were in a physically stable condition and provided written informed consent. The ethics committee of Canton Zurich, Switzerland, approved the study (Ref. Nr. EK: KEK-ZH-Nr. 2013-0286).

### Instrumentation: GAITRite® system

In order to assess temporal and spatial gait characteristics, the GAITRite® walkway analysis system (CIR Systems, Inc., Corporate Headquarters 376 Lafayette Ave. Suite 202, Sparta, NJ 0787) was used. It consists of a roll-up walkway (approximately seven meters long) with 13824 pressure sensors embedded in an active area of 366x61 cm, arranged in a grid-like pattern. Data were uploaded to a computer and automatic footstep identification took place. The system directly supplies clinicians and researchers with quantitative information about a subjects’ gait.

### Procedure

Subjects' characteristics, such as gender, age, height, weight, diagnosis, and Functional Gait Assessment (FGA) [11] score, were recorded. After informing and instructing the study participants about the measurement procedure, all participants completed one test to get them familiar with the setting and the GAITRite® system. Participants were advised to perform the measurement sessions wearing comfortable flat walking shoes and the same shoes were to be used for both measurements. The GAITRite® mat was positioned in a long and well-lit corridor. In order to assess the steady state of walking and to avoid recording the acceleration and deceleration phases, two meters of additional walking space before and after the mat allowed each participant room for starting and ending each walking trial [14, 38]. The patients and healthy subjects performed one walking trial for 7 protocol walking conditions. The same tester conducted all tests, operated the GAITRite® system and walked next to the participants to guarantee safety during the test.

The participants completed seven trials on the GAITRite® walkway following a predefined gait protocol. Prior to the trials, participants were given standardised instructions and a visual demonstration. The gait protocol was performed at a self-selected preferred walking speed and consisted of six tasks derived from the FGA in a non-random order [11] and with an additional cognitive task added to some of the walking trials.

The gait protocol included the following tasks of the FGA: [1] self-selected walking speed without dual task, [2] gait with horizontal head turns, [3] gait with vertical head turns, [4] gait with narrow base of support (with tandem steps), [5] gait with closed eyes and [6] walking backwards. In addition we tested gait with a dual tasking paradigm (counting backwards in steps of 7 from 100 during self-selected walking speed [39]). The latter task was added as we expected differences in temporal and spatial gait parameters between patients and healthy participants [40]. The FGA tests *Change in gait speed*, *Gait and pivot turn*, *Step over obstacle* and *Steps on stairs* where not recorded, as it was deemed not feasible or useful to be measured with the GAITRite® system.

Patients with vestibular disorders repeated the gait protocol after a ten-minute interval in order to assess the discriminant validity, the test re-test reproducibility and the measurement error. The following three temporal and spatial gait measurements were evaluated: Gait speed (m/s), cadence (steps/min.) and step length (cm). These outcome parameters were selected as they reflect disturbances in gait in patients with vestibular disorders [41]. The locomotion pattern of vestibular patients can, furthermore, be described with these three parameters [42]. These parameters are also sensitive to change and they improve after vestibular rehabilitation [3].

### Data processing

The recorded measurements were analysed immediately after each walking attempt on the GAITRite® system.

Footsteps, which did not fit completely within the active area of the GAITRite® system, were removed manually from the recorded walk. Mean values for each gait parameter were calculated. Further, in order to minimise environmental variability walking evaluations were conducted in the same hallway for each test.

### Statistics

Patients’ characteristics are described in Table 1. For hypothesis testing of the discriminative validity, unpaired *t*-tests were performed to determine the mean difference in measurements for gait variables in healthy subjects and patients with vestibular disorders.

**Table 1:** Subjects’ characteristics reported as mean values ± SD

	Healthy	Patients
	(*n* = 27)	(*n* = 35)
Female	13	14
Male	14	21
Age; years (SD)	44 (13)	59 (18)
Age range; years	25/70	18/86
Weight; kg (SD)	63 (21)	74 (15)
Height; cm (SD)	160 (47)	169 (9)
Score Funtional Gait Analyses SD)^a^	-	22 (6)
FGA range	-	3/30
- Bilateral peripheral vestibular dysfunction score	-	14
- Unilateral peripheral vestibular dysfunction	-	9
- Central vestibular dysfunction	-	6
- Morbus Menière	-	5
- vestibular migraine	-	1

^a^A cutoff score of 22/30 on the FGA provides optimum validity for classifying fall risk in older adults at risk for falling and in predicting unexplained falls in community-dwelling older adults. The FGA appears to predict falls in community-dwelling older adults better than the currently recommended clinical tools (e.g. Berg Balance Scale and the Timed Up and Go Test). This 22 from 30-possible-points cutoff score may be used as indicator for falls in elderly patients with vestibular dysfunctions [57]

ICC _(2.1)_ was used to determine test-retest reproducibility. For the interpretation of ICC values, benchmarks were used as described by Fleiss, Levin, and Paik [43] (>0.75 excellent, 0.40-0.75 fair-to-good, and <0.40 poor reliability). Since ICC only provides information about the reproducibility, the smallest detectable change [SDC] was additionally calculated at the 95 % confidence level using: $\mathrm{S}\mathrm{D}\mathrm{C}\kern0.5em =\kern0.5em 1.96\kern0.5em *\kern0.5em \mathrm{S}\mathrm{E}\mathrm{M}\kern0.5em *\kern0.5em \sqrt{2}$ [22]. The SEM was determined using $\mathrm{S}\mathrm{E}\mathrm{M}\kern0.5em =\kern0.5em \mathrm{S}\mathrm{D}\kern0.5em *\kern0.5em \sqrt{1\kern0.5em \hbox{-} \kern0.5em \mathrm{I}\mathrm{C}\mathrm{C}}$ [29, 44]. The SEM% as percentage of the mean (mean for the observations from test session 1 and 2) was defined by: SEM% = SEM/mean*100. The SDC% was calculated as the SDC divided by the mean for all measurements and multiplied by 100 % to be independent of the units of measurement [45]. The SDC% is a type of relative index and represents the limit for the smallest change that indicates a real change. A good measurement tool preferably shows low SEM and SDC values to be able to detect changes in a clinical trial [29].

Finally, systematic bias was assessed with the Bland-Altman analyses [46]. The Bland-Altman plot provides visual information in which the individual differences between the two measurements were plotted adverse to the individual means. The graph permitted the appraisal of the data regarding heteroscedasticity and detection of the minimal detectable change, which exceeds the measurement error in repeated measures [47].

Analyses were performed using SPSS Version 22.0 statistical software (SPSS, Inc. Chicago, IL) and level of significance was set at 5 %.

## Results

Thirty-nine outpatients were initially invited to participate in the study; four of these patients were subsequently excluded. Two patients were excluded due to being diagnosed with a benign paroxysmal positional vertigo, and diminished vertigo symptoms after treatment, one patient was excluded as he was younger than 18 years old and one patient was excluded due to the diagnosis of vestibular disorder not being able to be confirmed. The remaining 35 patients (21 men/14 women, mean age 59 ± 17 years, age range 18—86 years) were diagnosed with several vestibular disorders (bilateral peripheral vestibular dysfunction, unilateral peripheral vestibular dysfunction, central vestibular dysfunction, Menière’s disease, and vestibular migraine). The control group of 27 healthy adults (mean age 44 ± 12.7 years) consisted of 14 men and 13 women (Table 1).

For the parameter tandem walking, the GAITRite® software had considerable difficulty automatically detecting footfalls. Stolze, et al. [48] demonstrated that tandem walking in neurological patients consists of short and long steps, crossing of the legs and deviations of the foot from the ideal pathway. As missteps could not be recorded optimally with the GAITRite® system, tandem walking is not recommended as a valid test for assessing patients with vestibular dysfunction using the GAITRite® system. Thus, human intervention was required to process the data from two footfalls to one. As this is clinically not feasible, the measurements were declared invalid and not presented in the manuscript and tables.

The measurements of three patients yielded invalid values for negative step length and could not be used for the reproducibility analysis. Therefore, the analyses of 32 patients were performed for self-selected walking speed (see Table 3). Furthermore, for discriminant validity and reproducibility, one patient could not perform the task ‘walking under dual-task conditions, as he was afraid to loose balance or fall. These analyses were, therefore, performed with 34 patients (Tables 2 and 3).

**Table 2:** Discriminative validity between patients (*n* = 35) with vestibular disorders and healthy participants (*n* = 27) (determined by an unpaired *t*-test, level of significance at *p* ≤ 0.05)

	Gait speed (m/s)				Cadence (steps/min.)				Step length (cm)			
Gait tasks with;	Mean patient (SD)	Mean healthy (SD)	*p*	Mean Difference (CI)	Mean patient (SD)	Mean healthy (SD)	*p*	Mean Difference (CI)	Mean patient (SD)	Mean healthy (SD)	*p*	Mean Difference (CI)
Self-selected walking speed	1.2 (0.2)	1.4 (0.2)	<0.001	0.3 (0.2/0.4)	108.0 (10.4)	115.7 (8.0)	0.002	7.8 (3.0/12.6)	64.0 (9.9)	73.6 (9.4)	<0.001	10.0 (4.9/15.0)
Horizontal head turns	1.0 (0.2)	1.3 (0.2)	<0.001	0.3 (0.1/0.4)	98.8 (14.3)	107.2 (9.5)	0.010	8.5 (2.1/14.8)	60.8 (9.1)	71.2 (8.7)	<0.001	10.6 (5.9/15.4)
Vertical head turns	1.0 (0.3)	1.3 (0.2)	<0.001	0.3 (0.2/0.4)	99.5 (15.9)	109.8 (9.9)	0.004	10.3 (3.4/17.3)	60.0 (11.2)	71.6 (8.9)	<0.001	12.2 (6.7/17.6)
Closed eyes	0.8 (0.2)	1.2 (0.3)	<0.001	0.4 (0.2/0.5)	97.9 (15.0)	108.5 (13.6)	0.005	10.6 (3.3/18.0)	48.8 (11.8)	65.0 (10.6)	<0.001	15.0 (8.6/21.4)
Ambulating backwards	0.7 (0.2)	1.0 (0.2)	<0.001	0.3 (0.2/0.4)	95.8 (14.3)	105.1 (12.8)	0.010	9.2 (2.3/16.3)	40.8 (9.3)	54.4 (8.7)	<0.001	12.8 (8.2/17.4)
Dual task^a^	0.9 (0.2)	1.2 (0.3)	<0.001	0.3 (0.2/0.4)	88.3 (16.1)	101.8 (17.3)	0.005	11.4 (0.2/0.4)	58.8 (9.1)	68.4 (10.2)	0.001	8.8 (3.7/13.8)

^a^Dual tasking for Gait speed, Cadence and Step length was performed with *n* = 34^a^ patients. The dual-tasking paradigm was performed with a) self-selected walking speed and b) dual-tasking (counting backwards from 100 in steps of 7 during self-selected walking speed

**Table 3:** Reproduciblity and measurement error: ICC, SEM and SDC for patients with vestibular disorders (*n* = 35)

	Gait speed (m/s)			Cadence (steps/min.)			Step length (cm)		
Gait tasks with;	ICC (CI)	SEM	SDC	ICC (CI)	SEM	SDC	ICC (CI)	SEM	SDC
Self-selected walking speed	0.94	0.05	0.15	0.87	3.5	10.0	0.95^b^	2.2^b^	6.1^b^
	(0.89/0.97)			(0.76/0.94)			(0.91/0.98)		
Horizontal head turns	0.93	0.07	0.20	0.70	9.0	24.9	0.93	2.5	7.0
	(0.86/0.96)			(0.49/0.84)			(0.86/0.96)		
Vertical head turns	0.96	0.06	0.16	0.85	6.2	17.1	0.96	2.3	6.4
	(0.92/0.98)			(0.72/0.93)			(0.93/0.98)		
Closed eyes	0.87	0.08	0.23	0.83	6.3	17.3	0.92	3.4	9.4
	(0.78/0.94)			(0.70/0.92)			(0.85/0.96)		
Ambulating backwards	0.95	0.09	0.26	0.94	3.4	9.5	0.95	2.5	6.9
	(0.90/0.98)			(0.88/0.97)			(0.91/0.98)		
Dual task^a^	0.89	0.05	0.14	0.89^a^	5.6	15.5	0.87	3.4	9.6
	(0.79/0.95)			(0.79/0.94)			(0.75/0.93)		

^a^Dual tasking tests were performed with 34 patients; ^b^Step length during self-selected walking speed was performed in 32 patients. Three measures were invalid and could not be repeated. The dual-tasking paradigm was performed with a) self-selected walking speed and b) dual-tasking (counting backwards from 100 in steps of 7 during self-selected walking speed

### Hypothesis testing for discriminant validity of the GAITRite® walkway

Data for temporal and spatial gait parameters (gait speed, cadence and step length) and the specific walking conditions are presented in Table 2. For patients with vestibular disorders, the values in the 6 different walking tasks for gait speed varied from 0.7 to 1.2 m/s, for cadence from 88 to 108 steps/min. and for step length from 40 to 64 cm. See Table 2 for the mean values across all walking tasks. Data for gait speed varied for healthy control subjects from 1.0 to 1.4 m/s, for cadence from 101 to 115 steps/min. and for step length from 54 to 73 cm. All tests showed significant differences between the two groups (*p* ≤ 0.01) with differences generally above 10 % between patients and healthy participants for self-selected gait speed and step length. Patients with vestibular disorders walked more slowly in all test conditions with a lower cadence and a shorter step length compared to healthy controls.

### Reproducibility

**Fig. 1:**
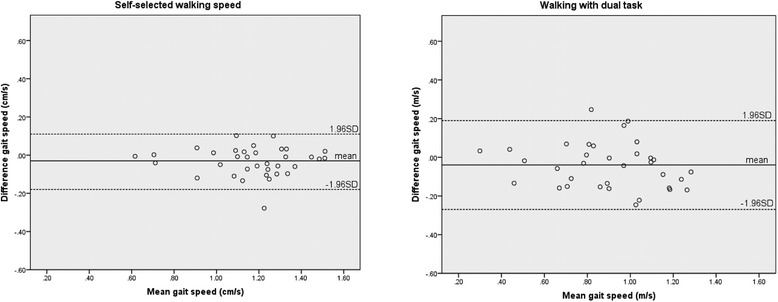
Bland-Altman plots for self-selected walking speed (left) and walking with dual task (right). Difference in individual self-selected gait speed between the test re-test sessions, plotted against the individual mean gait speed of the two sessions (m/s). The plot on the left side demonstrates self-selected walking, the plot on the right side self-selected walking speed with performance of a concurrent cognitive dual task. The mid line shows the mean difference (-0.03 m/s left and -0.04 m/s right), dashed lines show the upper and lower limits of (95 %) agreement (-0.18 and 0.11 m/s left and -0.27 and 0.19 m/s right)

Test re-test reproducibility of the walking tasks ranged with ICCs from 0.70 to 0.96 in patients with vestibular disorders. The range for the SEM for each gait condition varied from 2.5-9.0 %. The range for the SDC for all conditions was for the gait speed 0.14 to 0.26 m/s, for the cadence 9 to 24 steps/min. and step length from 6.4 to 9.6 cm (see Table 3). Most of the data were between 2 standard deviations in the Bland-Altman plots, with the exception of a few outliers (1-2) in gait speed and cadence for self-selected walking speed. The Bland and Altman plots for step length yielded 4 data points outside the 2 standard deviations. The Bland-Altman plots for gait speed, cadence and step length of the task self-selected walking speed for patients are illustrated in Fig. 1. The negative gait speed value of the mean difference line in the Bland-Altman plot indicates that the first walking attempt was generally slower than the second walking attempt. Lower cadence, as well as smaller step length values, were reported in the first walking session compared to the second (Fig. 1). Visual inspection showed no tendency towards heteroscedasticity.

## Discussion

The aim of this study was to evaluate the discriminant validity and re-test reproducibility of a walking assessment protocol measuring spatial and temporal gait parameters during different walking conditions and performed on the GAITRite® walkway analysis system.

The results of this study suggest that the walking assessment protocol performed on the GAITRite® system yields good discriminant validity between patients with vestibular disorders and healthy participants. Furthermore, excellent test re-test reproducibility values were determined for gait speed, cadence and step length measured under different walking conditions in patients with vestibular dysfunction.

### Discriminant validity

In this study, an approximate 10 % difference between patients and healthy controls in favour of healthy controls was observed in normal walking for the mean values of the parameters gait speed, cadence and step length. Differences for GAITRite® parameters between patients and healthy participants with a comparable magnitude were also measured in a report on individuals with cerebellar ataxia [48], patients with unilateral peripheral vestibular loss [UVL] and patients with bilateral peripheral vestibular loss [BVL] [41]. The small sample size and the heterogeneity of our sample (central vestibular dysfunction, M. Menière, and vestibular migraine) may have led to these differences. Conversely, other studies did not show significant difference for the parameters gait speed and cadence of self-selected walking between BVL patients and healthy controls [14, 17, 49]. However, the differences between groups in this study are supported by small SDC values that were smaller than the differences found between patients and healthy adults, thus indicating good discriminant validity of the protocol.

### Reproducibility and measurement error

The excellent ICC values found in this study are comparable with those of studies evaluating ICC in healthy elderly people [13, 14, 24], patients with a degenerative neurological disorder [50] and stroke patients [16, 25]. An improvement in gait parameters for patients with vestibular dysfunction after therapy can only be measured when the measurement error of the instrument is small enough to detect a real change [22]. A recently published review [3] described a clinical gait speed improvement of 0.2 m/s after vestibular rehabilitation in patients with UVL. Thus, the SDC values for self-selected walking found in this study were lower than those that might be expected following rehabilitation as revealed by Herdman [3]. To the best of our knowledge, comparable data are only available for the parameter gait speed in self-selected walking when determining the improvement on an individual patient level [3]. Parameters of measurement error will be more stable over different population samples than reproducibility parameters. Reproducibility parameters are highly dependent on the variation in the population sample and are only generalizable with samples of a similar variation. It is clearly a characteristic of the performance of an instrument in a certain group sample. Measurement error is more a characteristic of the measurement instrument itself. Measurement error parameters are preferable in all situations in which the instrument is used for evaluation purposes, which is often the case in medical research in a clinical setting. Researchers and clinicians should be eager to apply and interpret the parameters of measurement error (on an individual level) and reproducibility (on a group level) correctly [22].

To be of practical use, the results of the SDD should be interpreted as follows: when taking the measurement error into account, an SDC equal to or greater than 0.05 m/s (Table 3) between two measurements should be used as the threshold for a true clinical change in self-selected walking. In the Bland and Altman Plot for self-selected walking speed, the midline showed a mean difference between assessments of -0.03 m/s with a lower limit of (95 % agreement) of -0.18 and upper limit of agreement of 0.11 m/s, indicating that the patients walked slower in the re-test. The result of the other assessments in the reproducibility study should be interpreted in the same way (see Table 3, and Fig. 1 (self-selected walking with dual task).

The Bland-Altman plots showed a small systematic error between test and re-test (-0.03 m/s), albeit this difference did not reach significance. Therefore, it may be that patients became accustomed to the GAITRite® walking system and increased speed in their second attempt. Hamacher, et al. [51] recommended the application of a defined amount (familiarisation) of cycles to determine reliable measures of variability. The use of only one gait attempt that we used to familiarise the patients with the measurement set-up may influence the precision of variability measures. Although a familiarisation session was performed as previously proposed [52], the patients may have walked more confidently during the second test session. Training effects may explain the increase in gait speed during the second attempt [53] and the absence of severe vertigo symptoms in the participants. In order to minimize such a training effect and considering the time-restraints for assessments in the clinical setting we opted to conduct one test trial.

Further research should obtain comparable data for other gait parameters (cadence and step length) and in other walking conditions. Overall, the measurement protocol performed on the GAITRite® system indicates good reproducibility in patients with vestibular deficits and can, therefore, be recommended as a feasible assessment tool in the clinical setting.

### Different walking conditions

Walking speed decreased in patients with vestibular dysfunction while performing a dual task. Dual tasking also resulted in larger gait speed SDC values (23 to 26 %). It may be assumed that the task used in our study (counting backwards in steps of seven) affected the absolute reliability of gait speed more than a simple task would have, such as counting backwards in steps of two [49].

### Study limitations

This study has some limitations.

Firstly, the lack of a standardised measurement protocol for the GAITRite® system limits the interpretation of gait variability from evaluative and prognostic studies. The differences found in our study between patients with vestibular disorders and healthy subjects does not in itself prove validity for the GAITRite® system, it rather contributes to the evidence for or against validity which is an ongoing process. Further research is needed to standardise testing procedures and establish validity, reproducibility and measurement error for confident use of GAITRite® walking system in patients with vestibular disorders.

Secondly, the sample size was relatively small and may have affected the values of the reproducibility and measurement error. A sample size of at least 50 is generally seen as adequate for the assessment of the agreement parameter, based on a general guideline by Altman (1990) [54]. The sample size of 35 patients with vestibular disorders we used and 27 controls is, however, a realistic group size to find first estimates for the assumed relation between vestibular disorders and gait and to identify differences between patients and healthy controls.

Thirdly, the majority of vestibular patients reported that they did not have acute symptoms at the time of the test. Due to the chronic nature of vestibular disorders, most patients may have compensated their vestibular deficiency, e.g. by exaggerated hip sway in order to enhance balance or by looking down at the floor to avoid dizziness. This possibly influences the test. Furthermore, the group of healthy control subjects was younger than the group of patients with vestibular dysfunctions. Thus, the difference in gait speed found between patients and controls could depend on age in addition to the vestibular deficit [55].

Fourthly, the fact that we measured one walking trial per protocol walking condition may be regarded as an additional limitation. The amount of analysable gait cycles is limited because of this procedure and, in turn, influences the specificity of the gait measures [51]. However when using pressure walkways, stop and go movements introduce transients in the stride trajectories that have the potential to bias variability in terms of reproducibility and measurement error [51]. Conversely, in a clinical setting time and resource constraints often prevent performance of extensive measurement protocols. Furthermore, our patients performed their measures during clinical visits to the University Hospital and we did not want to daunt them with a stressful program.

Fifthly, the short time break of 10 min between measurements could influence the reproducibility and measurement error data in this study. However, one study reported good to excellent ICC’s (0.87-.097) for self-selected walking speed, cadence and step length and SEM’s with a 15-min break between measures [56]. Furthermore, the internal consistency of the FGA as determined with Cronbach alpha is with 0.79 [11] good, which further indicates that no behavioural response in gait is to be expected when gait is measured. Currently, there is no standardisation for an optimal time break between reproducibility measures. Thus, researchers and clinicians have to choose an optimal time frame when designing a study.

Despite these caveats, we believe that our study provides useful results regarding the reproducibility and measurement error measured with the 6 walking-conditions test performed on the GAITRite® system in patients with vestibular disorders. This documents the sustained deficit in gait patterns experienced by these patients when compared to healthy controls.

## Conclusions

The results of this study demonstrate that our walking protocol performed on the GAITRite® walkway analysis system results in valid and reproducible spatial and temporal gait parameters in patients with vestibular disorders. The addition of the GAITRite® system to clinical assessment protocols may determine a real change in gait speed, cadence and step length. The GAITRite® system may be employed in studies and clinical settings to determine the effect of disease outbreak and exercise in rehabilitation programs.

## Abbreviations

Cm: Centimetre
FGA: Functional gait assessment
ICC: Intraclass correlation coefficient
Kg: Kilogram
Min: Minute
*p*: Probability (*p*-value)
s: Seconds
SDC: Smallest detectable change
SD: Standard deviation
SEM: Standard error of measurement
VR: Vestibular rehabilitation
Y: Year
